# Sex Differences and Bone Metastases of Breast, Lung, and Prostate Cancers: Do Bone Homing Cancers Favor Feminized Bone Marrow?

**DOI:** 10.3389/fonc.2017.00163

**Published:** 2017-08-07

**Authors:** Mary C. Farach-Carson, Sue-Hwa Lin, Theresa Nalty, Robert L. Satcher

**Affiliations:** ^1^Department of Diagnostic and Biomedical Sciences, School of Dentistry, The University of Texas Health Science Center at Houston, Houston, TX, United States; ^2^Department of Translational Molecular Pathology, The University of Texas MD Anderson Cancer Center, Houston, TX, United States; ^3^Department of Orthopaedic Oncology, The University of Texas MD Anderson Cancer Center, Houston, TX, United States

**Keywords:** bone metastasis, prostate cancer, lung cancer, breast cancer, bone marrow, sex differences, sex hormones

## Abstract

Sex-associated differences in bone metastasis formation from breast, lung, and prostate cancer exist in clinical studies, but have not been systematically reviewed. Differences in the bone marrow niche can be attributed to sexual dimorphism, to genetic variations that affect sex hormone levels, or to the direct effects of sex hormones, natural or exogenously delivered. This review describes the present understanding of sex-associated and sex hormone level differences in the marrow niche and in formation of bone metastasis during the transition of these three cancers from treatable disease to an often untreatable, lethal metastatic one. Our purpose is to provide insight into some underlying molecular mechanisms for hormonal influence in bone metastasis formation, and to the potential influence of sexual dimorphism, genetic differences affecting sex assignment, and sex hormone level differences on the bone niche and its favorability for metastasis formation. We reviewed publications in PubMed and EMBASE, including full length manuscripts, case reports, and clinical studies of relevance to our topic. We focused on bone metastasis formation in breast, lung, and prostate cancer because all three commonly present with bone metastases. Several clear observations emerged. For breast cancer bone metastasis formation, estrogen receptor (ER) signaling pathways indicate a role for ER beta (ERβ). Estrogen influences the bone microenvironment, creating and conditioning a favorable niche for colonization and breast cancer progression. For lung cancer, studies support the hypothesis that females have a more favorable bone microenvironment for metastasis formation. For prostate cancer, a decrease in the relative androgen to estrogen balance or a “feminization” of bone marrow favors bone metastasis formation, with a potentially important role for ERβ that may be similar to that in breast cancer. Long-term estrogen administration or androgen blockade in males may feminize the bone marrow niche to one more favorable for bone metastases in prostate cancer. Administration of androgens in females, especially combined with mastectomy, may reduce risk of developing bone metastatic breast cancer. We conclude that it should be considered that females, those with female-leaning genetic variations, or hormonal states that feminize the bone marrow, may offer favorable sites for bone metastases.

## Introduction

During the past decade, it has become increasingly clear that a wide variety of molecular interactions in tumorigenesis and metastasis formation are influenced by sex and sex hormone level differences (1–7). Sex-specific differences in progression have been noted in several types of cancers that metastasize to bone including breast and lung. In contrast, the influence of sex determining factors and/or associated sex hormone administration, are logical factors to affect progression, but less well studied in prostate cancer patients receiving androgen blockade during treatment. For some cancers, the actions of sex hormones can promote or suppress tumor progression (7–10). Consequently, hormone levels, such as those of estrogens, progesterone, and androgens, often are therapeutically targeted in early stages of these cancers (5, 8, 11, 12). Some well characterized examples include estrogen receptor (ER)-positive breast cancer, where estrogens that promote tumor development are blocked therapeutically, and androgen-dependent prostate cancer, where androgen deprivation helps to suppress tumor progression. It is increasingly clear that the bone health of patients with bone metastases can be adversely affected by sex hormone deprivation therapy, resulting in treatment-related osteoporosis in breast and prostate cancer patients (8, 13, 14). The anticancer therapeutic survival benefits of such hormone therapies thus are balanced against the long-term potential for bone damage.

Bone is also frequently the site of metastatic disease. At present, 60–80% of patients with metastatic breast, lung, or prostate cancer develop bone metastases (8, 12, 15, 16). The complex bone marrow microenvironment provides a unique target for metastasis formation and subsequent tumor growth. Sex differences in the incidence and progression of bone metastasis have only recently become the focus of translational research. Consequently, endocrine pathways, including those regulated by estrogen, progesterone, or androgen, that are operative in bone and tumor biology have been identified to be key regulators of cancer bone cell cross-talk during the process of bone metastasis formation (12, 17, 18). Levels of sex hormones can vary widely in cancer patient populations, with differences owed to both endogenous factors such as genetics, and exogenous factors such as administration or inhibition of sex hormones.

We performed this systematic review using EMBASE and Pubmed databases to provide an overview of the potential mechanisms by which sex and sex hormone level differences influence bone metastasis formation. We included both basic and clinical studies from 1994 to 2017 that focused on the mechanism and incidence of bone metastases from three of the most common types of cancer that are influenced by sexual dimorphism and sex hormone levels, namely breast cancer, lung cancer, and prostate cancer. In our review of the literature, we sought answers to several questions about the relationships between sex, sex hormones, and genetic variations that influence sex hormone levels on the formation of bone metastases and the potential of these factors to condition the metastatic niche in bone to favor metastasis formation. We believe the answers to these questions will be relevant to clinicians and translational researchers interested in developing novel biologic therapies tailored to cancer patients with varying bone marrow states.

## Literature Analysis

Our review summarizes and integrates the findings of over 90 articles that addressed this subject. The results of our analyses are organized as a series of questions that we sought to answer by reviewing the existing literature and databases. Given the recent directive from the National Institutes of Health that sex as a biological variable be included whenever possible into basic, preclinical, and clinical studies (NOT-OD-15-102), this article is particularly timely in our opinion.

### Are Sex Hormone (Estrogen, Progesterone, Androgen) Driven Pathways Key Regulators of Bone Metastasis Formation?

Sex steroids were once considered to be the central regulators of bone metabolism in the aging skeleton, but increasingly, bone cell populations and various peptide hormones are recognized as being similarly influential (19). Key peptide hormones include leptin, receptor activator of nuclear factor kappa-B ligand (RANKL), parathyroid hormone, and the various fibroblast growth factors. Nonetheless, sex steroids remain key factors in musculoskeletal health for both males and females. Androgens and estrogens clearly influence the bone-forming osteoblast proliferation and the coupled osteoblastic stimulation of bone resorbing osteoclasts. Estrogens directly inhibit osteoclasts, and have the dominant effect on bone maintenance in both males and females. Androgens contribute directly to male periosteal bone expansion, mineralization, and trabecular bone maintenance (20–23); although the full spectrum of transcriptional targets of the estrogen, progesterone, and androgen receptors (ARs) in bone cells are not completely understood. Moreover, the effects of peptide hormones on bone resident cell populations are influenced by the prior conditioning of bone marrow by various levels and ratios of sex and steroid hormones. This interaction affects the way that bone cell populations perceive and respond to changes in levels of peptide hormones (24, 25). Taken together these studies suggest that sex hormone driven pathways play a significant role in bone metastasis formation, and that this is an area ripe for future systematic study.

### Does Sexual Dimorphism Impact Bone Metastases Formation?

It is increasingly clear that sexual dimorphism is an important factor for tumor growth, progression, and prognosis (3, 7, 8, 17, 18, 26). With primary tumors from breast, lung, and prostate cancers, sex and/or sex hormone-associated differences have been identified in numerous clinical studies (3, 6, 12, 14, 17, 27, 28). However, the underlying mechanisms that account for these differences have not been identified. By inference, evidence suggests that sex steroid hormones influence the pathogenesis of both primary tumors and bone metastasis formation, as well as physiological differences associated with genetic sex (3, 8, 15, 17, 28) or gender. For example, the phenotype of transsexuals with AR mutations and gonadal dysgenesis appears largely determined by sex steroid signaling regardless of chromosomal status (29). When there is interaction and overlap, such as in the case of postmenopausal or oophorectomized women with declining estrogen levels, or aging men with increased estrogen levels, much less is known and this subject remains to be investigated in more detail (30, 31). The literature on the relative influence of sexual dimorphism and genetic status versus sex steroid hormones on bone metastasis formation is limited, but is beginning to be the subject of translational work as is summarized in this review.

### What Is the Role of Estrogen in the Biology of Breast Cancer Bone Metastasis Formation?

Breast cancer can occur in both men and women. Bone is the most common site of metastasis, representing the first site of relapse for approximately 50% of patients (16, 18, 32). In contrast to prostate cancer, breast cancer more often metastasizes to other organs as well, with liver and/or lung involvement in at least 25% of patients, and high rates of central nervous system metastases (11, 33–35). Once breast cancer metastasizes to bone it is incurable, but often remains indolent (16). Moreover, the prognosis after the development of isolated bone metastases in breast cancer is significantly better compared with patients who also have non-osseous organ involvement (16, 28, 34).

Estrogens and their receptors play important roles both in the biology of breast cancer, and in the biology of bone. Estrogens exert their effects through two distinct receptors, estrogen receptor α (ERα) and estrogen receptor β (ERβ). Breast cancer cells and bone-forming cells or osteoblasts express both types of ERs, albeit at unique levels (28).

#### The Complex Association of Estrogen Receptor-Positive Breast Tumors with Bone Metastasis

Estrogen receptor positive (ER+) tumors are generally thought to have a good prognosis, but the relationship to bone metastasis formation is complex and incompletely understood. In the clinical literature, ER+ usually refers to the expression of ERα. Indeed, ERα has been the target for breast cancer treatment for years, whereas the role of ERβ is uncertain. However, there are some treatment resistant ER+ breast cancer tumors with poor prognosis and higher risk of bone metastases, implying that ERβ expression could be playing a role. Thus, the ongoing question is whether ER positivity, and specifically, ER+ α or β are associated with bone metastasis formation.

Clinical observations have identified an association between ERα positive breast tumors and the development of bone metastasis. Patients with ER+ (ERα) tumors have bone metastases three times more often than do patients with ER− tumors (28, 36). Relapse in bone is also more commonly associated with ER+ and/or progesterone receptor positive (PR+) tumors, suggesting a role for ERα in tumor progression associated with bone metastases (28). However, patients with isolated bone metastases have significantly prolonged survival compared with patients with additional non-osseous metastases, and form a clinically distinct group (28, 36). Complicating matters further, hormonal receptor status can be discordant between primary tumor and bone metastasis: 16–56% for ER and 14–44% for PR (36). However, the proportion of patients with isolated bone metastases that express ERα and/or PR is significantly higher than any other subset of breast cancer patients (36).

The molecular mechanisms responsible for bone metastasis formation are complex, and the influence of hormones is not well understood. Overall, the process involves several critical steps that are, in some cases, known to be regulated by sex steroids, and in others are possibly affected by biologic differences between male and female (8, 17, 18). The ER signaling pathways are implicated in disease progression (1, 26, 28, 30, 36, 37), because the ER status of bone metastases usually correlates with the ER status of the primary tumor (28, 36). Relatively little is known about the ER signaling pathways that lead to downstream activation of cellular targets that trigger disease progression; however, a protective role is generally suggested from limited clinical evidence: (1) Rabbani, et al. demonstrated a decrease in breast cancer cell growth and parathyroid hormone-related protein (PTHrP) production in response to estradiol (38); (2) in addition, overexpression of ER decreased tumor growth and reduced bone metastasis formation (38); (3) other studies have shown that kinase inhibitors that were designed to attenuate a host of pathways activated in breast cancer cells are not able to halt disease spread once multiple osseous metastases have appeared (16, 18, 28, 36, 39); and (4) a protective role of ERβ in the progression of breast cancer has been suggested, as higher levels of ERβ are predictive of positive response to the ER modulator tamoxifen (40, 41). The role of ERβ both in the primary tumor and in bone marrow where metastases occur has only recently become the focus for the development of treatments in addition to those that target ERα (40–42), but may provide an important second index by which to stratify risk in patients with ER positive breast cancers.

#### Estrogen-Responsive Genes in Bone Metastases

Similar to metastases that go to the brain or lung, there is a set of genes that favors breast cancer metastasis to bone (28, 33, 34). Wang et al. further studied and identified estrogen-responsive genes using an *in vitro* coculture system that modeled the colonization step of bone metastasis formation (28). The purpose of the study was to look for ER+ patterns associated with bone metastasis formation. A bone metastatic breast cancer cell line pair (parental line MDA-MB-231) stably expressing ERα or ERβ (MDA-ERα and MDA-ERβ) was cocultured separately with U2OS parental human osteoblastic cell lines that were similarly transfected (U2OS-ERα and U2OS-ERβ). Differences in gene expression were detected using microarrays. In breast cancer cells, 13 genes were identified that were altered solely by ERα, and 11 genes were found to be regulated solely by ERβ (28). Only five genes were regulated by both ERα and ERβ. Interestingly, in the bone cells the majority of genes were regulated by ERβ (only three genes for ERα and 13 genes for ERβ), suggesting that breast cancer cell–bone cell interactions are more likely regulated *via* ERβ. A gene expression signature associated with bone metastasis formation was identified (combined expression of Muc-1 and MacMarcks, regulated by ERβ) and verified with tissue samples from patients with infiltrating ductal carcinoma (28). These findings are consistent with other studies that implicate (43) ERβ regulation of Muc-1 in the pathogenesis of other adenocarcinomas (44–46); and MacMarcks, which belongs to a family of protein kinase C (PKC) substrates that have been shown to participate in cell adhesion (47). The estrogen responsiveness of these two gene products and their possible roles in the mechanism of breast cancer bone metastasis formation in the context of various genetic and hormonal states deserves further systematic study, especially in light of the observations that α and β ERs in bone cells antagonize one another (48).

Bone metastasis from breast cancer can appear decades after removal of the primary tumor(s). Latent bone metastasis formation likely depends on estrogen regulation, and occurs more frequently in breast cancer than many other types of cancer (16). The majority of late onset metastases occur as osteolytic lesions in bone. Zhang et al. showed that the rate of late onset bone metastasis, identified as a relapse after 5 years from cancer diagnosis, was significantly higher in ER+ cases (16). A gene expression signature denoting Src activity in the tumor was shown to be tightly associated with latent bone metastasis formation (16). Src mediates protein kinase B, also known as Akt, a key factor in the regulation of cancer cell survival in the bone metastasis microenvironment.

### How Do Sexual Dimorphism and Chromosomal Differences Affect Bone Metastasis Formation in Breast Cancer?

Differences in breast cancer bone metastases formation have been observed between males and females (1, 3, 8, 26, 30, 36, 49). Possible mechanisms for sexual dimorphism include: (1) anatomical differences in men that increase the likelihood that a tumor can access the circulation and metastasize via a hematogenic pathway (1); (2) a relative paucity of breast tissue in men compared to women, and close tumor proximity to skin and nipple that facilitates dermal lymphatic spread (37); (3) effects of reproductive cycle on carcinogenesis (26); (4) biological differences in the tumors themselves (1, 30, 37); and (5) differences in the sex hormonal status of the bone cells at sites of metastases (50).

Manifestations of these sex based differences result in:

(a) The limited benefit of anti-hormonal treatment in men despite the overall higher rate of ER/PR expression compared with female breast cancer patients.
(b) The lack of influence of nodal stage for male breast cancer patients compared with females.
(c) The predominant influence of T-stage on the observed poorer overall survival for men with breast cancer compared to women, and higher local recurrence rates (1).
(d) Overall inferior prognosis for male breast cancer patients compared with stage matched females.

Falk et al. performed one of the few studies exploring the influence of sexual dimorphism on bone metastasis formation and bone pain (3). An animal model for bone metastasis was used that introduced mammary cancer cells into the femoral cavity of both female and male BALB/cJ mice. Interestingly, the female mice had earlier onset of tumor growth and associated bone pain compared with male mice. Moreover, the estrous phase did not influence tumor growth in female mice. However, there was no difference observed for the extent of bone degradation (3); and, the differences in overall disease progression in bone dissipated over time. Overall, the study supports the hypothesis that the female bone marrow differs from that in males and provides a more advantageous microenvironment for metastatic colonization (3).

#### Breast Cancer and Bone Metastasis Pathophysiology in Transsexual Patients

Transsexual and transgender patients with breast cancer are a unique cohort to consider the relative influences of genetics versus sex hormones. An excellent recent review of cancer risk in this patient population at seven sites was recently published (51). Male-to-female (M to F) transsexual/gender patients typically receive oral estrogens for prolonged periods in order to maintain secondary female characteristics. Growing numbers of breast cancer have been reported in these transsexual/gender (M to F) women (52) and in the residual breast tissue of transsexual (F to M) men (53). Some, but not all, are estrogen dependent breast carcinomas. One case study reported a 41-year-old M to F patient receiving estrogen therapy for 14 years with a symptomatic tender lump in the left breast and no family history of breast cancer. A 13 mm triple-negative grade 3 invasive ductal carcinoma was diagnosed (54). Another recent report suggested that breast cancer in M to F transsexuals occurs at a younger age and is likely to be more ER negative than is breast cancer in a comparable group born biologically male, but only studied 10 individual cases (55). Gooren et al. studied a larger cohort documenting the occurrence of breast cancer in 2,307 M to F transsexual Dutch patients, and concluded that the risk of breast cancer development in transsexual/gender women was not increased by hormone administration (56). Breast cancer incidences were comparable with male breast cancers and, therefore, lower than in the female population (56). Their findings support the hypothesis that M to F subjects have similar risk as the natal sex, i.e., male breast cancer; and female-to-male (F to M) individuals have similar risk as the new sex, i.e., male breast cancer, as the risk may be greatly reduced by the combination of mastectomy and testosterone treatments in these patients (49, 52, 56). The role of BRCA1 in trans-women is unknown as we were unable to find any studies or case reports of breast cancer in reviewing the literature, although a case of a BRCA1 positive M to F opting to forego mastectomy was reported (52). A published case study (57) described a M to F transsexual BRCA2 positive patient with recurrent disease who developed breast cancer after 7 years of cross-sex hormonal therapy without being aware of being a member of an established BRCA2 mutation-positive kindred. This issue of awareness in the trans community also is highlighted by a breast cancer study conducted by the Veterans Health Administration (1996–2013) (58, 59). In a cohort of 5,135 trans individuals, 11 breast cancer cases were reported that included seven birth sex females and four birth sex males. Three of the birth sex males presented with late-stage disease that was fatal, whereas most of the birth sex female F to M veterans presented with earlier stage disease that was treatable. Examination of the cohort as a whole did not suggest that the incidence of primary breast cancer is higher in trans veterans.

Whether hormonal treatments used in M to F sex reassignment favor bone colonization is unknown, and deserves further study. It is intriguing to think about whether “feminization” of bone marrow by estrogen administration influences patterns of metastasis to bone, rather than the incidence of cancer in breast tissue. The transsexual/gender population receiving long-term hormonal therapy may provide a cohort in which to ask if this is the case, and whether such patients should be monitored more closely for bone metastasis if a primary cancer diagnosis is made, especially if maintenance with female hormones is continued.

#### Breast Cancer in Patients with Klinefelter Syndrome

Klinefelter syndrome is a non-inherited chromosomal condition associated with hypogonadism in males who possess an extra copy of the X chromosome and are hence 47, XXY (60). Compared with normal 46, XY men, adults with Klinefelter syndrome live with an increased risk of osteoporosis and of developing breast cancer (60). A 3,518 patient cohort study that evaluated the risk of cancer in men diagnosed with Klinefelter syndrome showed that the standard mortality ratio (SMR) for breast cancer compared to a normal XY males was 57.8, 95% CI = 18.8–135 (60). In this same study, the standardized mortality ratios were found to be especially high for men with 47, XXY mosaicism who developed breast cancer (SMR = 222.8, 95% CI = 45.9–651.0). It is not known if the bone marrow of these men is “feminized” by the presence of an extra X chromosome that favors bone colonization, and this would be an interesting area to investigate. Likewise, the impact of feminizing chimerism in bone marrow for males who are XY/XY^−^ or XY/XX, which can occur constitutively or after bone marrow allograft transplant, on bone metastasis is completely unexplored, but is expected to present similar risks to those with Klinefelter.

For the future, many important questions remain regarding the influence of sexual dimorphism versus sex hormones on formation of bone metastases in the diverse disease we collectively call breast cancer. The role of estrogen ablation or blockade in promoting epithelial to mesenchymal transition with invasion (61), and in homing of breast cancer cells to bone, needs to be further studied as a sexually dimorphic variable, as does the potential for feminization of bone by long term estrogen administration that may favor bone metastasis. Bone metastases of breast cancer remain incurable, and the existing approaches for treatment such as bisphosphonates are non-specific, and do not account for the sex of the patient regardless of the primary tumor of origin (8). Identification of sex hormonal and sex-dependent mechanisms and differences in target molecules will potentially allow the identification of new diagnostic and therapeutic strategies for bone metastatic breast cancer.

### What Is the Role of Estrogen and Androgen in Lung Cancer Bone Metastasis Formation?

Several recent studies have reported on sex differences in the incidence and mortality of human lung cancer, including a delayed increase and leveling off of lung cancer risk in women in comparison to men (5, 6, 27, 62). Moreover, the therapeutic response to chemotherapy is more favorable in women (27). However, the mechanism by which sexual dimorphism and sex hormones influence lung cancer bone metastasis formation is less well studied than for breast cancer (6, 27, 63, 64).

#### Sexual Dimorphism and Lung Cancer Bone Metastasis

To understand the potential role of sexual dimorphism in lung cancer bone metastasis formation, it is useful to first review the sex differences for primary tumors in the lung. Numerous studies have observed that sex steroids and their receptors are important mechanisms underlying sex differences, particularly with non-small cell lung cancer (NSCLC). Expression and regulation by ERs have been reported in both lung cancer cell lines and human lung carcinoma specimens (63). Niikawa et al. showed that the tissue concentration of estradiol in NSCLC is higher than in normal lung tissue, and that estradiol increased the proliferation of a NSCLC cell line that stably expresses ERα (65–67). Martinez et al. also showed that ER+ cells acquire a more aggressive phenotype than ER− cells when cultured on an extracellular matrix produced by a bone cell line (68). Anti-estrogen treatment also has been reported to induce anti-proliferative effects in NSCLC cells, and combined treatment with growth factor receptor inhibitors, including gefitinib and erlotinib, further reduced growth (69).

More recently, studies have focused on pathway interactions between growth factor receptors and ER signaling in lung cancer. A similar strategy was used previously for breast cancer, and is the basis for combined targeted therapy. A study by Nishio et al. showed that the epidermal growth factor receptor (EGFR) inhibitor, gefitinib, suppressed serum androgen levels; moreover, gefitinib responders had significantly lower androgen levels than non-responders in female NSCLC patients (70–72). Kerr et al. used laser capture microdissection of tumor specimens to analyze NSCLC gene expression, showing that both ERα and ERβ mRNA transcripts are expressed at higher levels in NSCLC cells compared with normal lung (63). In addition, microarray data showed that ERα expression was associated with differential gene expression in fewer than 20 genes compared to normal lung cells; whereas ERβ expression modulated more than 500 genes (63). An inverse relationship also was observed between ERβ and EGFR mRNA levels, analogous to previous observations reported for breast cancer with ERβ and EGFR protein expression (63).

Sakaguchi et al. recently reported the results of one of the only translational studies that focused on sex differences in bone metastasis from small cell lung cancers (SCLC) (6). Human SCLC cell lines expressing ERβ and AR, but not ERα, were injected into the tail veins of severe combined immunodeficiency (SCID) mice depleted of natural killer cells. The formation of multiple organ metastases was compared between male and female mice, using prostate cancer PC3 cells and human breast cancer MCF7 cells as controls. Interestingly, the number of bone metastasis from lung cancer cell lines was increased in female mice compared to males. There was no difference in the observed number of metastases to the lungs or liver (6). This increase in bone metastasis formation also could be produced by androgen blockade or castration of male mice (6), suggesting an important role for sex steroids. This difference could be by alteration of the biology of the cancer cells themselves, but also could be due to the feminization of the bone marrow by androgen loss. Huang et al. (73) reported that bone marrow-derived mesenchymal stem cells (MSCs) subjected to androgen depletion displayed enhanced self-renewal, indicating that the AR has a repressive role in stem cell expansion. Another intriguing explanation could be the sex-influenced differences in the nature of the MSCs present in the bone niche. For example, it has been reported that female MSCs secrete more pro-angiogenic factors compared to male MSCs (74). Whether this plays a role in promoting metastasis and successful bone colonization, however, has not been reported.

#### PTHrP May Affect Lung Cancer Bone Metastasis Formation

The PTHrP expression patterns in lung cancer might involve a sex-related mechanism that contributes to differences in bone metastasis formation, although this has not been proven. Tumor PTHrP is associated with increased survival in patients with NSCLC in a sex-dependent manner (27, 75, 76). Women with tumors who displayed PTHrP immunoreactivity have better survival than women with tumors negative for PTHrP. This effect was not observed in men, suggesting a sex-related mechanism for the pro-survival effect in women (75, 76). Using an orthotopic model for NSCLC (expressing ERβ and AR but not ERα), Montgrain et al. reported that tumor burden was lower in female mice than male mice (27). Lung tumors in females expressed more PTHrP than seen in males. This finding was possibly due to negative regulation of PTHrP by androgen in male mice (27). Finally, Miki et al. identified a role for PTHrP in bone metastasis formation, demonstrating that PTHrP produced by human SCLC cells contributes to the development of bone metastases, but not visceral metastases, in their SCID mouse model (64). The role of sex was explicitly tested using the same SCID model in later work by Sakaguchi et al., as described above (6, 64). Similar to breast cancer, lung cancer bone metastases formed nearly twice as often in female mice using tumor cells that express PTHrP and ERβ, and once again point to a potential role for feminized bone marrow in supporting growth of bone, but not soft tissue, metastases.

### What Is the Role of Sex Steroids in Prostate Cancer Metastasis to Bone?

The influence of androgens and estrogens on the development of prostate cancer and bone metastasis is complex and not well studied (10, 77). Zhau et al. demonstrated that the androgen-repressed state, which occurs with a relative increase in estrogen levels, was positively associated with prostate cancer progression and metastasis formation, similar to the lung cancer study (78); leading to the hypothesis that a decrease in the androgen/estrogen ratio with aging or therapy could be responsible in part for prostate carcinogenesis, rather than higher absolute blood levels of steroid hormones (10). Therapeutically, estrogen and androgen blockades have been commonly used for treatment of advanced prostate cancer, although the mechanisms remain incompletely known. In an *in vitro* study using PC3 and 22Rv1 PCa cells conducted by Dey et al., it was shown that ERβ1 inhibited proliferation and factors known to be involved in bone metastasis; whereas ERβ2 had increased proliferation and upregulated factors involved in bone metastasis (77).

#### Prostate Cancer in Transsexuals

To gain additional insight about the role of sex versus hormones in the biology prostate cancer, it is interesting to review the literature on prostate cancer incidence in M to F transsexuals. Many years before presenting with PCa, these patients receive hormone ablation as part of their sex reassignment therapy (79). Thus, their disease is already defined as castrate resistant at the time it is initially diagnosed. In our review of the literature, we could only find one manuscript that reported on a cohort of such patients. Gooren et al., report on 2,306 M to F patients, who all had been orchiectomised and treated with estrogens (80). There was a single case of PCa identified in the group, indicating that PCa is extremely rare in these patients. A total of four cases have been reported in additional case reports (79, 81–83). There were three that described bone metastasis. Turo et al. describe a representative case of a patient that developed PCa at the age of 75, after undergoing M to F sex reassignment surgery at age 45 (79). The patient died at age 80 from a thromboembolic event after starting chemotherapy for progression of osseous metastatic disease (79). These findings indicate that it will be important to distinguish clinically between the risk of initially developing prostate cancer in this cohort, which appears to be low, from the risk of developing rapidly progressing disease once a bone metastasis has occurred in the context of feminized bone marrow, which may be significantly higher.

#### Extra Female Chromosome with Reduced Testosterone and Prostate Cancer

For men with Klinefelter syndrome, the mortality for prostate cancer was found to be reduced (SMR = 0, 95% CI = 0–0.7), suggesting a protective effect from the reduced levels of testosterone owed to the extra X chromosome (60). Endocrine disruption also comes with an increased risk of dying from lung cancer. Compared to the general population, men with Klinefelter syndrome had a higher mortality from lung cancer (SMR = 1.5, 95% CI = 1.0–2.0) (60). It would be interesting to examine these effects in men with mosaic forms of Klinefelter syndrome that have 47, XXY lung or prostate and normal 46, XY bone marrow; or with normal 46, XY lung or prostate and 47, XXY bone marrow; but to our knowledge this has not been done.

#### Impact of Sexual Dimorphism on Bone Metastasis Treatment with Bisphosphonates and RANKL Inhibitors

Randomized controlled trials that demonstrate efficacy have been completed in both men and women for all commonly used osteoporosis drugs, including alendronate, risedronate, zoledronate, ibandronate, denosumab, and strontium ranelate (84–88). In the case of males receiving androgen deprivation therapy, randomized controlled trials have demonstrated the effectiveness of pamidronate, alidronate, resedronate, zoledronic acid, denosumab, and the selective ER modulators raloxifene and toremifene (89). RANKL inhibition *via* denosumab administration is used to treat bone metastases in both breast and prostate cancer patients. It has been reported that RANKL expression inversely correlates with a metastatic phenotype (90–92). A decrease in autocrine RANKL stimulation might trigger RANK expressing tumor cells to be attracted by RANKL expressing osteoblasts in bone, and foster cancer homing to the marrow (93).

Sexual dimorphism in the RANKL pathway is unlikely. A recent meta-analysis of genome-wide bone mineral density association studies identified no significant sexual dimorphisms (94). However, some evidence for sexual dimorphism was reported recently in a study examining how the lipid raft protein caveolin is regulated by RANKL. Cav-1 expression is induced by RANKL during osteoclastogenesis. Moreover, this RANKL-induced osteoclastogenesis and subsequent bone resorption is blocked by Cav-1 inhibition *via* knockdown or silencing. A sexual dimorphism was observed in mice, where Cav-1-deficient female mice, but not male mice, were shown to be osteopetrotic. Cav-1-deficient female mice had higher bone volume and trabecular volume compared to wild type mice, with increased trabecular number and reduced trabecular separation. The Cav-1-deficient male mice had higher osteoclast numbers, and bone volumes similar to wild-type mice. The female phenotype was attributed to reduced osteoclast differentiation, and was shown to be restored by Cav-1 overexpression. Overall, Cav-1 deficiency affected the maturation of osteoclast precursor cells in a manner dependent on the sex of the mice, although the mechanism of this was not determined. Cav-1 has been suggested to regulate ER trafficking to the cell membrane. Estrogen has a proapoptotic effect on osteoclasts. ERα deletion suppresses this effect in females, but not males, so that the Cav-1 effect might be due to a stronger dependency of estrogen signaling on Cav-1 in females (95). Sexually dimorphic effects of ER and Cav-1 and bone cancer metastasis are worthy of further mechanistic study.

## Summary and Discussion

This study provides an overview of the influences of sex differences and sex hormones in the development of bone metastasis from breast, lung, and prostate cancers. A summary of the influence of sex-related risk factors on bone metastasis formation and their association with sexual dimorphism is shown in Table 1. Overall, this is an area of translational research that is in its early stages, and remains highly worthy of future work as we move toward precision medicine for metastatic diseases.

**Table 1:** Intrinsic factors present in primary tumors and/or bone/bone marrow that increase risk of bone metastasis formation.

Risk factor	Primary tumor	Bone/bone marrow	Dimorphic^a^
Sex steroid status	Steroid resistance	Androgen blockade	Yes
Sex steroid receptors	AR(−), ER(+), PR(+)	ERα, β(+)	Yes
Genetic sex	?^b^	XX, XXY, XY–	Yes
Src	Activated	?	?
Muc-1	Positive	N/A	?
MacMarcks	Positive	N/A	?
RANKL	Positive	Positive	No

*^a^Yes: a demonstrated association with sexual dimorphism has been reported in the literature. For details see text. No: a lack of association with sexual dimorphism has been reported in the literature. Those with “Yes” should be considered as a biological variable in study design*.

*^b^?: unresolved issue in the literature*.

In summary, studies of the ER signaling pathways have implicated a role for ERβ in females for promoting colonization of the bone microenvironment by breast cancer cells, along with subsequent metastasis formation and spread (11, 16, 18, 28, 40). The molecular mechanisms may include downstream estrogen regulation of Muc-1, MacMarcks, a PKC substrate, the sarcoma family member Src, or EGFR. Sex differences have been reported in breast cancer bone pain, progression, and cancer growth in bone, including work by Falk et al. that demonstrated a more favorable bone microenvironment for breast cancer metastasis in female mice (1, 3, 4, 26, 37). Future translational studies are needed to further explore the relevant interactions and cross-talk between metastasizing cells and cells native to the bone tumor microenvironment that are regulated by sex hormones.

Translational studies focused on NSCLC (5, 27, 63–66, 69–72, 76) support the hypothesis that females have a more favorable bone microenvironment for metastasis formation. Similar to breast cancer, inhibiting the EGFR has been used to treat lung cancer patients (14, 17, 27). The lung cancer results with mice suggest differences attributable to sexual dimorphism (6). More work is needed to answer the question of why bone metastases form more readily in female mice, and to determine the mechanisms by which bone marrow feminization fosters the growth of bone metastases.

Additionally, there is a clear role for ERs in promoting bone metastasis formation in prostate cancer (10, 14, 77, 78). The estrogen/androgen balance plays a role in cancer disease progression in patients with genetic alteration (XXY) or sex reassignment hormonal treatment (60, 79–83). However, the responsible mechanisms for both of these phenomena at the cellular level remain unclear.

### Are There Sex-Related Differences in Metastases of Other Cancers?

Hormone levels do not completely account for sex-related differences with all cancers. For example, lung cancer and brain cancer show clear sex-related differences in disease progression (5–7, 35, 63). Primary brain tumors occur more frequently in males, and males suffer worse outcomes from brain tumors than females (7). This disparity in brain tumorigenesis is present regardless of age or sexual maturity and, therefore, is not a consequence of the acute effects of circulating sex hormones. Brain metastases occur in a third of all adult cancer cases, and also appear to be influenced by sex, occurring more frequently in males (7). Although lung cancer rates are nearly equivalent in men and women in the US (54% male), most brain metastases from lung cancer occur in male patients (58–83%) (7). The same disparity has been observed for melanoma derived brain metastases, which occur disproportionately in males, suggesting that sexual dimorphism in biological functions including underlying genetics, immune function or tumor microenvironment may be responsible (8). Unlike bone, evidence does not support the notion that feminization of brain tissue makes it a more favorable site for metastasis.

## Conclusion

**Figure 1:**
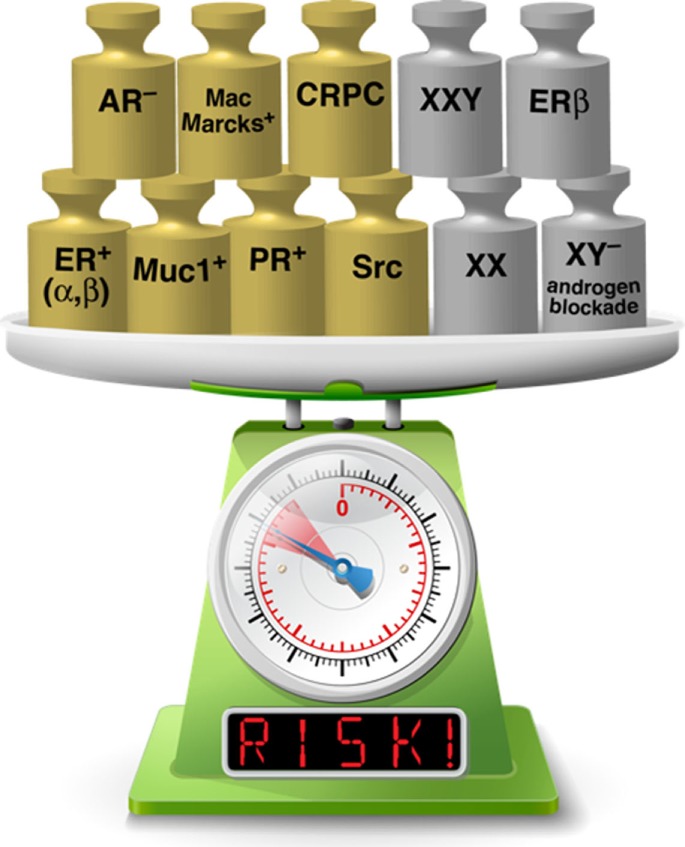
Factors present in both cancer cells and bone metastatic sites increase risk of developing lethal bone disease. Gold weights indicate factors present in cancer cells and silver weights represent factors present in cells in the bone tumor microenvironment (See text for more details). Feminization of the bone marrow environment may add to risk in the same way that factors present in the bone metastatic cancer cells are known to do [Image of scale modified from an image licensed from klyk@123RF.com].

Sex-based differences in the development of bone metastasis have been observed in both preclinical and clinical studies, and are most likely attributable to a combination of hormonal regulation and underlying biology related to sexual dimorphism. The concept that feminization of bone marrow by administration or blockade of sex steroids can alter outcomes is highly worthy as an area for future study, both *in vitro* and *in vivo*. Figure 1 is a visual representation of factors in bone metastatic cancer cells themselves, and those that feminize bone marrow, such that the balance of the scale may tip to favor bone colonization and development of lethal disease.

In our opinion, the most immediate gap that would benefit from immediate attention is examination of the role of ERβ in the preferential development of bone metastasis. Currently, there is one selective ERβ antagonist available, PHTPP, which has 36-fold selectivity over ERα. It is a synthetic, non-steroidal molecule that has been used in preclinical scientific research. Clinically, there are no agents developed and no studies that have been reported. In contrast, there are numerous ERβ agonists (3b-diol, 8b-VE2, phytoestrogen, diarylpropionitrile) that could be tested in preclinical work, and possibly later in clinical trials. Alternatively, selective agonists of ERα or ERβ could be tested and developed. The available selective antagonists are much more limited, including MPP (methylpiperidinopyrozole), but these could provide promising new leads for new therapeutics targeting bone metastases.

More work delineating the role of the genetic sex of the bone marrow in the promotion of bone metastasis formation needs to be undertaken to develop new strategies for targeting treatments to the individual. The most obvious cancers to examine are breast and lung cancer. One could design a clinical trial using agents that prevent the feminization of bone marrow microenvironment in order to prevent or dampen metastasis formation. It would also be interesting to discover if the sex of metastasizing cells influences the relevant interactions that lead to bone metastasis formation. Preclinical studies are required in order to identify specific targets for potential treatments that could be applied in a sex-specific fashion. Asking and answering these questions would be consistent with the goals of the NIH in understanding sex as a biological variable that can impact treatment planning and decision-making.

## Author Contributions

MCF-C and RS cowrote the article, performed literature search, and are responsible for content presented and conclusions drawn. S-HL reviewed the manuscript, participated in discussions to be drawn from literature search, and contributed substantially to conclusions reached. TN assisted with literature search, drafted parts of text, and contributed to interpretation of literature.

## Conflict of Interest Statement

The authors declare that the research was conducted in the absence of any commercial or financial relationships that could be construed as a potential conflict of interest.
